# Perioperative corticosteroids for preventing complications following facial plastic surgery

**DOI:** 10.1590/1516-3180.20141325T2

**Published:** 2014-07-22

**Authors:** Edina Mariko Koga da Silva, Bernardo Hochman, Lydia Masako Ferreira

## Abstract

**BACKGROUND:**

Early recovery is an important factor for people undergoing facial plastic. However, the normal inflammatory processes that are a consequence of surgery commonly cause oedema (swelling) and ecchymosis (bruising), which are undesirable complications. Severe oedema and ecchymosis delay full recovery, and may make patients dissatisfied with procedures. Perioperative corticosteroids have been used in facial plastic surgery with the aim of preventing oedema and ecchymosis.

**OBJECTIVES:**

To determine the effects, including safety, of perioperative administration of corticosteroids for preventing complications following facial plastic surgery in adults.

**METHODS:**

*Search strategy:* In January 2014, we searched the following electronic databases: the Cochrane Wounds Group Specialised Register; the Cochrane Central Register of Controlled Trials (CENTRAL) (The Cochrane Library); Ovid MEDLINE; Ovid MEDLINE (In-Process & Other Non-Indexed Citations); Ovid Embase; EBSCO CINAHL; and Literatura Latino-Americana e do Caribe em Ciências da Saúde (LILACS). There were no restrictions on the basis of date or language of publication. *Selection criteria:* We included RCTs that compared the administration of perioperative systemic corticosteroids with another intervention, no intervention or placebo in facial plastic surgery. *ata collection and analysis:* Two review authors independently screened the trials for inclusion in the review, appraised trial quality and extracted data.

**MAIN RESULTS:**

We included 10 trials, with a total of 422 participants, that addressed two of the outcomes of interest to this review: swelling (oedema) and bruising (ecchymosis). Nine studies on rhinoplasty used a variety of different types, and doses, of corticosteroids. Overall, the results of the included studies showed that there is some evidence that perioperative administration of corticosteroids decreases formation of oedema over the first two postoperative days. Meta-analysis was only possible for two studies, with a total of 60 participants, and showed that a single perioperative dose of 10 mg dexamethasone decreased oedema formation in the first two days after surgery (SMD = -1.16, 95% CI: -1.71 to -0.61, low quality evidence). The evidence for ecchymosis was less consistent across the studies, with some contradictory results, but overall there was some evidence that perioperatively administered corticosteroids decreased ecchymosis formation over the first two days after surgery (SMD = -1.06, 95% CI:-1.47 to -0.65, two studies, 60 participants, low quality evidence). The difference was not maintained after this initial period. One study, with 40 participants, showed that high doses of methylprednisolone (over 250 mg) decreased both ecchymosis and oedema between the first and seventh postoperative days. The only study that assessed facelift surgery identified no positive effect on oedema with preoperative administration of corticosteroids. Five trials did not report on harmful (adverse) effects; four trials reported that there were no adverse effects; and one trial reported adverse effects in two participants treated with corticosteroids as well as in four participants treated with placebo. None of the studies reported recovery time, patient satisfaction or quality of life. The studies included were all at an unclear risk of selection bias and at low risk of bias for other domains.

**AUTHORS' CONCLUSIONS:**

There is limited evidence for rhinoplasty that a single perioperative dose of corticosteroids decreases oedema and ecchymosis formation over the first two postoperative days, but the difference is not maintained after this period. There is also limited evidence that high doses of corticosteroids decrease both ecchymosis and oedema between the first and seventh postoperative days. The clinical significance of this decrease is unknown and there is little evidence available regarding the safety of this intervention. More studies are needed because at present the available evidence does not support the use of corticosteroids for prevention of complications following facial plastic surgery.

**This is the abstract of a Cochrane Review published in the Cochrane Database of Systematic Reviews 2014, issue 6, Art. No.: CD009697. DOI:** 10.1002/14651858.CD009697.pub2 (http://cochrane.bvsalud.org/cochrane/main.php?lib=COC&searchExp=Perioperative%20and%20corticosteroids%20and%20for%20and%20preventing%20and%20complications%20and%20following%20and%20facial%20and%20plastic%20and%20surgery&lang=pt). For full citation and authors' details, see reference 1.

**The abstract is available from:** http://onlinelibrary.wiley.com/doi/10.1002/14651858.CD009697.pub2/abstract.

## References

[1] da Silva  EM, Hochman  B, Ferreira  LM (2014). Perioperative corticosteroids for preventing complications following facial plastic surgery. Cochrane Database Syst Rev.

